# Mandatory Laparotomy in Penetrating Abdominal Injuries with Omental Evisceration: Experience in a Major Trauma Center in the Philippines

**DOI:** 10.7759/cureus.5688

**Published:** 2019-09-18

**Authors:** Marie Shella B De Robles, Eduardo C Ayuste Jr

**Affiliations:** 1 Surgery, Philippine General Hospital, Manila, PHL

**Keywords:** omental evisceration, penetrating trauma, stab wound, therapeutic laparotomy

## Abstract

Background: Omental evisceration due to abdominal stab injuries connotes peritoneal penetration and translates to around 70% risk of intra-abdominal injury. Such cases are being managed with mandatory laparotomy at the Philippine General Hospital. This study aims to review the patient profile and laparotomy outcomes in such cases.

Methods: This is a retrospective review of 98 consecutive laparotomies performed for patients with omental evisceration secondary to abdominal stab wounds between January 2004 to April 2018.

Results: Almost all patients were male (99%) with a mean age of 32.1 years (range 14-70). The majority (81%) had a therapeutic laparotomy, and only 19 patients (19%) had a non-therapeutic laparotomy. The most commonly injured organs include the small bowel, stomach, colon, diaphragm, and liver. There was no significant difference in age, sex, duration of injury, systolic blood pressure and heart rate at presentation between the two groups. There were significantly more patients who presented with peritonism in the therapeutic laparotomy group compared to the non-therapeutic laparotomy group (82% vs 53%, p=0.005). Patients who presented with peritonism were six times more likely to have a therapeutic laparotomy. There was no significant difference between morbidity and mortality rates in the two groups. The length of hospital stay for the non-therapeutic laparotomy group was significantly shorter compared to the therapeutic laparotomy group (3.6 vs 5.7 days, p=0.006).

Conclusion: The rate of therapeutic laparotomy remains to be significantly higher among patients with omental evisceration. Hence, omental evisceration, particularly those associated with peritonism, should continue to prompt operative management.

## Introduction

Abdominal stab injuries with omental evisceration represent 7% of all laparotomies performed for penetrating abdominal injuries in the Philippine General Hospital (PGH). Omental evisceration connotes peritoneal penetration and translates to around 68%-75% risk of intra-abdominal injury [1]. Such cases are managed with mandatory laparotomy at PGH. Recently, selective non-operative treatment has become more popular than routine laparotomy due to higher rates of unnecessary exploration. 

While organ evisceration is an indication for laparotomy, omental evisceration is not considered as an absolute indication and non-operative management may be considered in selected cases. In a study by Arikan, the overall incidence of patients who had no significant abdominal pathology after routine exploration for penetrating abdominal stab wounds with organ or omentum evisceration was 54.1% (33/61) [2]. The overall incidence of significant injuries among asymptomatic patients was 36.5% (19/52) [2].

The purpose of this study is to review the patient profile and laparotomy outcomes of patients with omental evisceration due to penetrating abdominal injuries managed in our institution.

## Materials and methods

This was an observational study using trauma registry data at the PGH. PGH is a major trauma center situated in Manila, Philippines. The hospital treats around 1500 major trauma cases and performs more than 500 trauma operations in a year

The records of all patients with omental evisceration secondary to abdominal stab wounds managed in PGH over ten years from January 2008 to April 2018 were retrospectively reviewed. Patients with sutured wounds from a different institution and no apparent evisceration at the time of consultation at PGH, despite notifications of omental prolapse elsewhere, were excluded. The patients were classified into two groups depending on the intra-operative findings during exploration. Patients with no intra-abdominal pathology and those with solid organ with no imminent danger to the patient’s life or posing no hemodynamic instability were classified as non-therapeutic laparotomy, and injuries that threatened the life or deranged hemodynamic stability unless surgically managed were classified into the therapeutic laparotomy group.

Statistical analysis was performed using SPSS version 23.0 (IBM, Armonk, New York, USA). All variables are expressed as mean values. Categorical data are presented as number and percentage and were compared with Fisher’s exact test or the Chi test as appropriate. Continuous variables were compared using Student’s t-test as appropriate. Logistic regression analysis was performed to identify independent predictors of therapeutic laparotomy. Statistical significance was set at *p* < 0.05 for all analyses.

This study was approved by the University of the Philippines Manila Research Ethics Board (UPMREB) Review Panel. The UPMREB granted a waiver of consent. This study was a review of records concerning non-sensitive data and constituted a study of minimal risk. No foreseeable risks are involved, and no direct benefits are associated with this study.

## Results

The demographic details of the patients are shown in Table 1.

**Table 1:** Demographic and post-operative details of patients undergoing laparotomy for omental evisceration secondary to abdominal stab wounds ^a^Unpaired two-tailed t test performed for continuous variables. ^b^Two-tailed Fisher’s exact/Pearson-Chi square test performed for categorical variables.

	Overall (N = 98)	Therapeutic laparotomy (N = 79)	Non-therapeutic laparotomy (N = 19)	P value
Age (years), mean + SD^b^	32.1 + 11.6	32.2 + 11.4	31.7 + 12.5	0.89
Sex (Male:Female)^a^	97:1	78:1	19:0	1.00
Duration of injury (hours), mean + SD^b^	3.9 + 3.5	4.0 + 3.8	3.4 + 2.1	0.46
Systolic blood pressure (mmHg), mean + SD^b^	107.7 + 21.2	106.9 + 22.6	111.1 + 13.7	0.44
Heart rate (beats/minute), mean + SD^b^	90.9 + 10.8	90.9 + 11.4	90.6 + 8.6	0.92
Peritonism on presentation^ a^	75 (77%)	65 (82%)	10 (53%)	0.005
Morbidity rate^ a^	1 (1%)	1 (1%)	0	1.00
Mortality rate^a^	5 (5%)	5 (6%)	0	0.58
Length of hospital stay (days), mean + SD^b^	5.2 + 2.9	5.7 + 3.0	3.6 + 1.8	0.006

**Figure 1:**
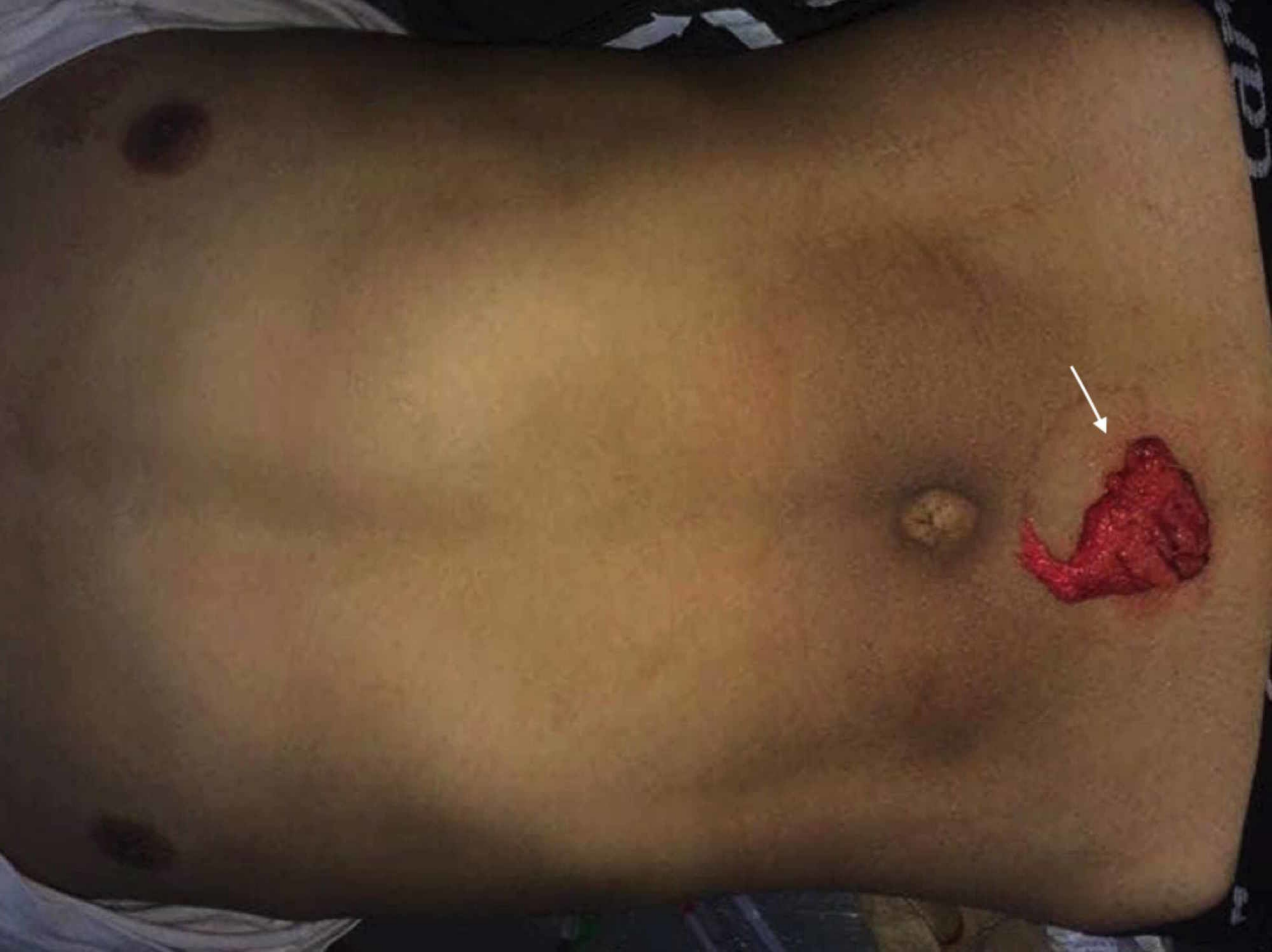
Patient presenting with omental evisceration (white arrow) after anterior abdominal stab wound

Between January 2004 to April 2018, laparotomy was performed in 98 consecutive patients with omental evisceration secondary to abdominal stab wounds. (Figure 1)

Almost all patients were male (99%) with a mean age of 32.1 years (range 14-70). As an initial approach, the standard resuscitative protocols approved by the Advanced Trauma Life Support (ATLS) guidelines were followed. Preoperative antibiotics with Cefuroxime 1.5 g and Metronidazole 500 mg were administered intravenously in all patients. The mean interval from the time of injury to the presentation at the PGH emergency department was 3.9 hours (range 0.25 to 23).

The majority of the patients (79/98, 81%) had a therapeutic laparotomy, and only 19 patients (19%) had a non-therapeutic laparotomy. Among those who had a therapeutic laparotomy, 54% (43/79) had only one injured organ discovered intra-operatively, and the remaining 46% (36/79) had two or more organs injured. The most commonly injured organs include the small bowel, stomach, colon, diaphragm, and liver. (Table 2)

**Table 2:** Most commonly injured intra-abdominal organ noted during laparotomy

	n
Jejunum/Ileum	34
Stomach	20
Colon	14
Liver	14
Diaphragm	11
Spleen	10
Duodenum	9
Pancreas	8
Kidney	4
Major vessel	4
Gallbladder	2

The overall morbidity rate was 1%, and the overall mortality rate was 5%. The mean length of hospital stay was 5.2 days (range 1-18).

There was no significant difference in age, sex, duration of injury, systolic blood pressure, and heart rate at presentation between the therapeutic laparotomy and non-therapeutic laparotomy groups. (Table 1) Meanwhile, there were significantly more patients who presented with peritonism in the therapeutic laparotomy group compared to the non-therapeutic laparotomy group (82% vs 53%, P = 0.005). Patients who presented with peritonism were six times more likely to have therapeutic laparotomy (Table 3).

**Table 3:** Analysis based on Cox-model regression

Variable	Coefficient	Standard error	Hazard ratio	P value
Age (years)	-.013	.024	.987	0.58
Duration of injury (hours)	.029	.079	0.972	0.72
Systolic blood pressure (mmHg)	-.001	.012	.999	0.94
Heart rate (beats per minute)	-.006	.027	.994	0.83
Peritonism on presentation	1.782	.610	5.944	0.003

All 19 patients who had non-therapeutic laparotomy did not have any significant morbidity or mortality. Meanwhile, the mortality rate for the therapeutic laparotomy group was 6% (5/79). Four of these patients died intra-operatively not only because of the severity of the injury acquired but also because of the delay in consultation. Two of these patients sustained abdominal aortic injuries, while the other two had severe splenic injuries associated with massive blood loss that was not promptly managed due to delay in consult. The fifth patient died of severe sepsis secondary to an anastomotic leak from the previous duodenal injury repair performed on the initial abdominal exploration. The length of hospital stay for the non-therapeutic laparotomy group was significantly shorter compared to therapeutic laparotomy group (3.6 vs. 5.7 days, P = 0.006).

## Discussion

Trauma literature describes a category of laparotomies that reveal no pathologic findings as “non-therapeutic.” Non-therapeutic laparotomy is also defined by some as laparotomy for a minor injury that in retrospect, might not have required surgical treatment [3]. The reported incidence of non-therapeutic laparotomy for trauma varies from 1.7% to 38%, depending on the experience and practice patterns of the individual trauma center [3]. Although the site of penetration may provide clues as to which organs could be injured, the location of entry alone does not accurately predict organs at risk. In the presence of omental or bowel evisceration, severe abdominal injuries may be found in as much as 75% of patients, with half of these having more than one intra-abdominal organ injury [1]. These results are consistent with the findings of this study.

Peritonitis and hemodynamic instability constitute strong indications for emergency laparotomy. Hence, initial vital signs and abdominal physical examination on admission remains the cornerstone of trauma triaging. The incidence of significant intra-abdominal injury approaches 85% for hemodynamically stable patients with signs of generalized peritonitis [4]. However, patients with short pre-hospital transport times may not immediately exhibit signs or symptoms of shock or peritonitis despite the presence of significant internal bleeding or organ injury.

Although most injuries will declare themselves on initial clinical assessment, there is a small but significant group of patients with normal vital signs and physical examination that may have an occult injury that if missed can cause serious problems. Many screening modalities are available to investigate such cases further. However, the utility of these tests are limited in our institution and sometimes do not assist much in decision-making. Plain radiographs of the abdomen are not useful in patients with stab wounds to the anterior abdomen unless there is suspicion of an embedded foreign body. Some studies examining patients with abdominal stab wounds found that an overwhelming majority of patients with injuries requiring repair had a normal abdominal radiograph [5-6]. Computed tomography scan of patients with abdominal stab wounds identifies solid organ injury with high accuracy (100% sensitivity, 96% specificity) and evaluates the retroperitoneum well [5-6]. It does not, however, detect peritoneal penetration and is reported to be unreliable in the detection of bowel and diaphragmatic injuries. Therefore, it should not be used routinely for the assessment of patients with anterior abdominal stab wounds.

Physical examination also has significant limitations in certain situations. Patients with polytrauma, particularly those with concomitant severe head or spinal injury, may be difficult to assess. Alcohol or other substances that may result in altered sensorium may affect the accuracy of clinical assessment. Not only do combative and intoxicated patients pose a diagnostic dilemma due to the lack of reliable physical examination and potential danger to healthcare personnel. Also, these patients tend to be uncooperative, particularly during imaging studies, which require the patient to remain still. Thus, the surgeon must have a high index of suspicion for the presence of possible injuries in order not to miss them.

Unnecessary laparotomy should be avoided if possible. Whereas non-operative management carries the inherent risks of a missed hollow visceral injury, delayed bleeding, and transfusion-related risks, laparotomy also carries a different set of risks [7-8]. The reported incidence of laparotomy or anesthesia-related early complications varies between 8.6% and 25.6% [3]. The incidence of late complications like bowel obstruction from postoperative adhesions and incisional hernia is reported to be between 2.4% and 5% [3]. Both the overall cost of hospitalization and length of hospital stay for patients undergoing therapeutic laparotomy are also significantly higher compared to patients successfully managed non-operatively [3]. Prudent judgment should be exercised in deciding non-operative management in penetrating abdominal trauma. This strategy requires close monitoring and frequent application of diagnostic tools to detect significant intra-abdominal injury promptly. It should be used cautiously in centers with limited trauma resources.

## Conclusions

The rate of therapeutic laparotomy remains to be significantly higher among patients with omental evisceration. Hence, omental evisceration, particularly those associated with peritonism, should continue to prompt operative management. Prospective studies may be needed to investigate further the role of selective non-operative management for patients with a benign abdominal examination to provide a more accurate and appropriate recommendation for this subset of patients.
